# Human Astroviruses: A Tale of Two Strains

**DOI:** 10.3390/v13030376

**Published:** 2021-02-27

**Authors:** Virginia Hargest, Amy E. Davis, Shaoyuan Tan, Valerie Cortez, Stacey Schultz-Cherry

**Affiliations:** 1 Department of Infectious Diseases, St. Jude Children’s Research Hospital, Memphis, TN 38105, USA; virginia.hargest@stjude.org (V.H.); amy.davis2@stjude.org (A.E.D.); shaoyuan.tan@stjude.org (S.T.); valerie.cortez@stjude.org (V.C.); 2 Department of Microbiology, Immunology, and Biochemistry, University of Tennessee Health Science Center, Memphis, TN 38163, USA

**Keywords:** human astrovirus, HAstV-1, VA1, viral replication, barrier permeability, nitazoxanide

## Abstract

Since the 1970s, eight closely related serotypes of classical human astroviruses (HAstV) have been associated with gastrointestinal illness worldwide. In the late 2000s, three genetically unique human astrovirus clades, VA1-VA3, VA2-VA4, and MLB, were described. While the exact disease associated with these clades remains to be defined, VA1 has been associated with central nervous system infections. The discovery that VA1 could be grown in cell culture, supports exciting new studies aimed at understanding viral pathogenesis. Given the association of VA1 with often lethal CNS infections, we tested its susceptibility to the antimicrobial drug, nitazoxanide (NTZ), which we showed could inhibit classical HAstV infections. Our studies demonstrate that NTZ inhibited VA1 replication in Caco2 cells even when added at 12 h post-infection, which is later than in HAstV-1 infection. These data led us to further probe VA1 replication kinetics and cellular responses to infection in Caco-2 cells in comparison to the well-studied HAstV-1 strain. Overall, our studies highlight that VA1 replicates more slowly than HAstV-1 and elicits significantly different cellular responses, including the inability to disrupt cellular junctions and barrier permeability.

## 1. Introduction

Human astroviruses (HAstVs) are positive-sense single-stranded RNA viruses. Epidemiological studies have shown that “classical” HAstVs, which include eight distinct serotypes (HAstV-1-8), are a leading cause of pediatric viral gastroenteritis worldwide and approximately 90% of children possess detectable antibodies to at least one serotype by the age of five [1,2,3,4]. In addition to the “classical” HAstV strains, two genetically distinct “non-classical” HAstV clades were identified in 2009 [5,6], collectively known as Virginia/Human-mink-ovine (VA/HMO) viruses and are more genetically related to animal astroviruses than the classical HAstVs [7,8].

Among all HAstV strains, HAstV-1 is the most prevalent worldwide [9,10,11,12]. While the prevalence of the non-classical VA viruses has been difficult to assess due to inadequate testing, recent seroprevalence studies suggest significant exposures to VA1 in at least 65% of adults [13,14]. Unlike the classical HAstVs, the association of VA strains with gastroenteritis is unresolved [15,16]. However, it is clear that VA viruses can cause systemic infections that involve the respiratory tract [17] and central nervous system (CNS) [18,19]. To date, there have been 10 reported cases of HAstV-induced CNS infections, half of which were associated with VA1 infection [18,19,20,21,22,23,24,25,26]. Closely residing in the same clade as VA1, mink and ovine astroviruses have also been associated with CNS disease [27,28,29] potentially indicating that these particular members of the *Mamastrovirus* genus have distinct molecular and cellular mechanisms of disease in comparison to classical HAstV. 

Of the 10 CNS-related HAstV infections, more than half have been fatal [18,20,21,23,25,26], highlighting the need for effective treatments. Recently, our lab demonstrated that the antimicrobial drug nitazoxanide (NTZ) inhibits the replication of multiple classical HAstV by blocking double-stranded RNA (dsRNA) formation [30]. Given the recent development of VA1 strains that could replicate in cell culture [31,32], we asked if VA1 was similarly susceptible to NTZ. Like the classical HAstVs, VA1 replication was inhibited by NTZ treatment. We were quite surprised that NTZ could be added to VA1 infected cells for much longer times post-infection compared to HAstV-1 suggesting that VA1 replication kinetics may differ. Thus, in these studies we explored VA1 replication kinetics and impact on Caco-2 cells. Our data demonstrate that VA1 replicates more slowly than HAstV-1 and fails to disrupt cellular junctions or epithelial barrier permeability suggesting that VA1 pathogenesis is unique from the classical HAstVs.

## 2. Materials and Methods

### 2.1. Cells and Virus Propagation

The human intestinal adenocarcinoma Caco-2 cell line was obtained from ATCC (HTB-37). Cell propagation was performed in complete growth media composed of minimum essential medium (MEM; Corning) supplemented with 20% fetal bovine serum (FBS; HyClone, Logan, UT, USA, 1 mM sodium pyruvate (Gibco ThermoFisher, Waltham, MA, USA), and Glutamax-I (Gibco ThermoFisher). Caco-2 cells were allowed to differentiate by seeding cells on 6.5 mm semipermeable transwell inserts (polyester membranes, 0.4 µm pore size; Corning) at 2.5 × 10^4^ cells per well and incubated for 5 to 7 days until the transepithelial electrical resistance (TER) measurements read greater than 1000 Ω.

Human Astrovirus-1 (HAstV-1) was propagated in Caco-2 cells for 3 to 4 days in infection media consisting of 0.3% bovine serum albumin (BSA; Gibco), 1 mM sodium pyruvate, and Glutamax-I in MEM. The cells were then harvested and underwent 3 to 4 freeze-thaw cycles before quantitating viral titer by fluorescent focus assay (FFA) as previously described [33]. 

VA1 astrovirus propagation was performed as described [34]. Briefly, Caco-2 cells were infected with VA1 in complete Caco-2 growth media for 5 to 7 days. Cells were harvested and freeze-thawed followed by titer determination by FFA in Caco-2 cells.

### 2.2. Nitazoxonide (NTZ) Treatment

Nitazoxanide (Sigma-Aldrich) was reconstituted according to manufacturer’s specifications to 10 mM in DMSO, subsequent dilutions were carried out in serum-free media. In vitro treatment with NTZ was performed as previously described [30]. Briefly, 5 × 10^4^ cells were seeded into 96-well tissue culture plates (Corning, Tewksbury, MA, USA), and after 2 days, the cells were inoculated with VA1 (MOI of 2) in serum-free MEM for 1 h at 37 °C. Following adsorption, the inoculum was removed and at the indicated times 2.5 µM NTZ or vehicle alone (DMSO) was added. Infection proceeded until 24 h post-infection (hpi) when cells were fixed, stained for viral capsid and the percent of capsid positive cells quantitated.

### 2.3. Capsid and dsRNA Staining

Following infection, cells were washed with 1× phosphate-buffered saline (PBS; Corning), fixed with 100 µL 4% paraformaldehyde (ThermoFisher, Waltham, MA, USA) for 20 min at room temperature and permeabilized for 15 min with 0.05% Triton X-100 (Sigma Aldrich, St. Louis, MO, USA). Viral capsid was detected by immunofluorescent microscopy as previously described [35]. Briefly, fixed monolayers were blocked for 1 h at room temperature with PBS containing 5% normal goat serum (NGS; Gibco). Cells were then incubated with anti-dsRNA (J2; Scicons, Szirák, Hungary), anti-HAstV-1 mouse monoclonal antibody 8e7 (Invitrogen ThermoFisher, Waltham, MA, USA) or anti-VA1 rabbit monoclonal antibody (generously provided by Dr. David Wang, Washington University School of Medicine in St. Louis, St. Louis, MO, USA) diluted in 1% NGS for 1 h at room temperature. Cell monolayers were then washed 3 times with 1× PBS and incubated with anti-mouse or anti-rabbit IgG labeled with either Alexa Fluor 488 or 555 (Invitrogen) secondary antibody and with Hoescht stain (ThermoFisher) at room temperature for 30 min. Imaging was performed on the EVOS FL cell imaging system followed by analysis with ImageJ 1.50i software.

### 2.4. Viral Kinetics

Caco-2 cells seeded in 96-well tissue culture plates (Corning) were incubated until confluency, at which time they were mock-infected or infected with HAstV-1 or VA1 (MOI of 2). Following adsorption for 1.5 h, inoculum was removed, and cells were washed twice with PBS. Serum-free media was replaced on HAstV-1-infected cells and growth media was replaced on VA1-infected cells. At the indicated time points, cells were washed with 1× PBS, fixed, and stained for viral capsid and double-stranded RNA (dsRNA) by immunofluorescent staining. The percent of dsRNA and capsid positive cells was then quantitated.

### 2.5. p-ERK Western Blotting

Caco-2 cells were mock infected or infected with HAstV-1 or VA1 (MOI of 10). At the indicated times, cells were lysed in 1× RIPA Lysis Buffer (Millipore Sigma Aldrich, St. Louis, MO, USA) containing 1X protease and phosphatase inhibitor cocktail (Pierce ThermoFisher, Waltham, MA, USA) for 15 min at room temperature. Equal protein concentrations of the soluble fraction were separated by sodium dodecyl sulfate-polyacrylamide gel electrophoresis (SDS-PAGE) (4-20%) under reducing conditions. Following transfer to nitrocellulose membrane and blocking in Tris-buffered saline containing 0.05% Tween 20 (TBST; ThermoFisher) and 5% bovine serum albumin (BSA; Sigma Aldrich), membranes were probed with rabbit anti-pERK1/2 or anti-ERK1/2 (diluted 1:1000 in TBST) for 2 h at room temperature. The blot was imaged on LiCor Odyssey Fc and band densitometry was measured using Image Studio version 5.2 software. To ensure equal loading, blots were probed for total β-actin as described above.

### 2.6. U0126 Treatment

In vitro treatment with U0126 was performed as previously described [36]. Briefly, 5 × 10^4^ cells were seeded into 96-well tissue culture plates (Corning), and after 2 days, the cells were preincubated with 10 μM U0126 (Promega, Madison, WI, USA) or vehicle alone (DMSO) for 1 h prior to infection. The cells were then inoculated with VA1 (MOI of 2) in serum-free MEM for 1 h at 37 °C. Following adsorption, the inoculum was removed and 10 µM U0126 or vehicle was added. Infection proceeded until 24 hpi when cells were fixed and stained for the presence of viral capsid. The percent of capsid positive cells was quantitated ad compared to no treatment.

### 2.7. Cytokine Analysis

Caco-2 cells were mock infected or infected with HAstV-1 or VA1 (MOI of 0.1). At the indicated times, cell supernatants were collected and 25 μL was analyzed in duplicate by the 13-plex LEGENDplex Human Anti-Virus Response Panel (BioLegend, San Diego, CA, USA) on a BD LSR Fortessa. 

### 2.8. Cytotoxicity Assay

Cytotoxicity was measured using the CellTiter-Glo 2.0 Cell Viability Assay (Promega). Briefly, Caco-2 cells were mock infected or infected with HAstV-1 or VA1 (MOI of 10). At 24 hpi, CellTiter-Glo 2.0 Reagent was added to the cells and supernatant at a 1:1 ratio. The solution was mixed to allow for cell lysis and incubated for 10 min at room temperature. Luminescence was measured on a Cytation 5 Cell Imaging Multi-Mode Reader (BioTek, Winooski, VT, USA).

### 2.9. Occludin Staining

Briefly, Caco-2 cells were seeded onto glass coverslips. Once confluent, the cells were infected with VA1 (MOI of 10) or mock infected. At 24 hpi, cells were fixed with 100% ice cold methanol and then blocked with 5% normal goat serum (NGS) in PBS at room temperature for 1 h. The cells were stained for occludin (71-1500; Invitrogen). The coverslips were then mounted with Prolong Gold Antifade Mountant (Invitrogen) and sealed. Cells were imaged with a Zeiss LSM 780 NLO mounted on inverted Axio Observer microscope. Images were captured with a 60X oil-immersion objective lens using Zeiss ZEN software.

### 2.10. Transepithelial Electrical Resistance (TER)

TER values for differentiated Caco-2 cells were monitored with a voltohmmeter (CellZscope+; NanoAnalytics, Münster, Germany), which was placed inside of an incubator set to 37 °C and 5% CO_2_. Following a baseline reading, cells were infected HAstV-1 or VA1 at an MOI of 1 or 10 or mock-infected. After the 1 h adsorption period, the inoculum was removed and measurement of TER was carried out in real-time every 15 min for 24 h.

### 2.11. Capsid Structure Analysis

The reference protein sequence Astrovirus VA1 capsid (accession number ASJ26376.1) was downloaded from Genbank; and HAstV-1 reference (CP core domain 5EWN and spike domain 5EWO [37]) was fetched in PyMOL (Version 2.0 Schrödinger, LLC). PyMOD3.0 [38] was used for the following analysis. Alignments of ASJ26376.1 with sequences of 5EWN and 5EWO were created separately using ClustalW [39]. VA1 capsid sequences were trimmed according to the alignment. Using 5EWN and 5EWO crystal structure as reference, homology modelling was performed with the trimmed VA1 sequences using MODELLER package [40]. Pairwise conservation analysis was performed to mark similarity of VA1 capsid protein sequences with HAstV-1 reference. Sequences were marked based on their conservation as follows: residues that are perfectly conserved (red), residues that are not perfectly conserved but that have a BLOSUM62 score higher than 0 (pink) and residues with a BLOSUM62 score lower than 0 or residues that are aligned to a gap in the reference (white). VA1 capsid protein core and spike domain crystal structures were then visualized in PyMOL.

## 3. Results

**Figure 1:**
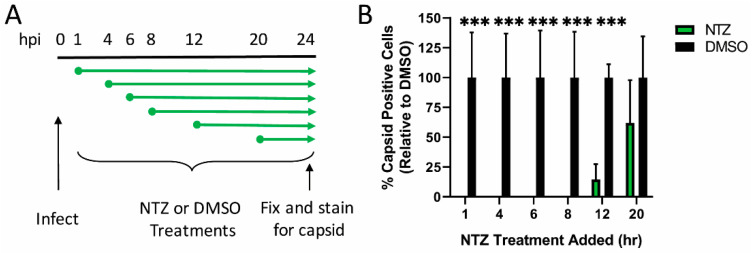
Nitazoxanide effectively inhibits VA1 replication when added up to 12 h post-infection. (**A**) At the indicated times post-infection, nitazoxanide (NTZ; 2.5μM) was added to VA1-infected Caco-2 cells. At 24 hpi, cells were fixed and stained for the presence of VA1 capsid protein. (**B**) The percent of VA1 capsid positive cells for each NTZ treatment was quantitated and compared to vehicle alone (DMSO). All error bars indicate standard deviation of two combined, independent experiments performed in triplicate, and asterisks show statistical significance as measured by multiple t tests as follows: ***, *p* < 0.001.

We previously showed that the nitazoxanide (NTZ) inhibits the replication of multiple classical astrovirus strains when added up to 8 h post-infection (hpi), corresponding to a block in the formation of dsRNA [30]. To determine if NTZ also inhibits VA1, Caco-2 cells were infected with VA1 at an MOI of 2 and treated with 2.5 μM of NTZ or vehicle control at 1, 4, 6, 8, 12, and 20 hpi. NTZ completely inhibited VA1 capsid formation when added up to 8 hpi, with a significant reduction still observed when added at 12 hpi (Figure 1). The addition of NTZ at 20 hpi showed no significant difference from the addition of vehicle alone. These data highlight that NTZ is a potent antiviral drug against VA1. The fact that NTZ is effective even when added at longer times post-infection suggests that the VA1 replication cycle may be slower than HAstV-1. 

**Figure 2:**
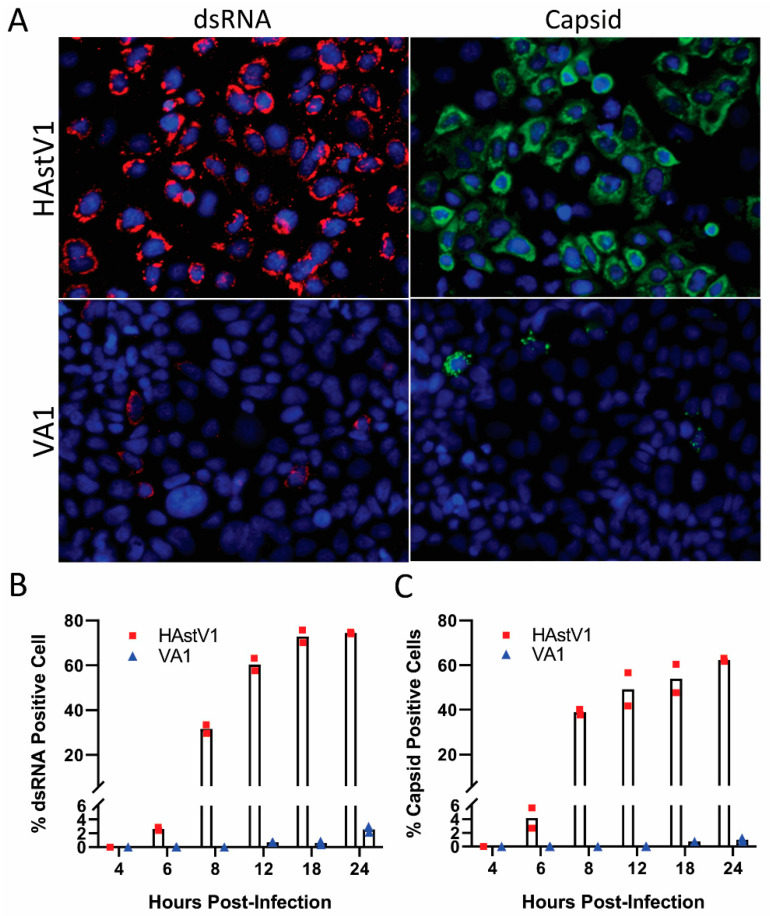
VA1 replication is delayed in comparison to HAstV-1. Caco-2 cells were mock-infected or infected with HAstV-1 or VA1 (MOI of 2). At various time points, cells were stained for viral capsid and double-stranded RNA (dsRNA) by immunofluorescent staining. (**A**) Representative images, taken at 40× magnification on EVOS FL, of HAstV-1 and VA1 capsid production and dsRNA formation at 24 hpi. The percent of dsRNA positive (**B**) and capsid positive (**C**) cells at the indicated timepoints was quantitated.

To examine the replication kinetics of VA1, we analyzed double-stranded RNA (dsRNA) formation and capsid production over a 24-h time course in comparison to HAstV-1, using an MOI of 2 (Figure 2). HAstV-1-infected cells had detectable levels of both dsRNA and capsid protein at 6 hpi. In contrast, we did not observe dsRNA in VA1-infected cells until 12 hpi and capsid staining did not occur until 18 hpi (Figure 2B). By 24 hpi, HAstV-1 capsid was detected in nearly 75% of cells. In contrast, only approximately 2.5% of cells were positive for VA1 at 24 hpi (Figure 2C). These data suggest there is at least a 6-h delay in the start of VA1 replication and the subsequent replication kinetics are delayed compared to HAstV-1.

**Figure 3:**
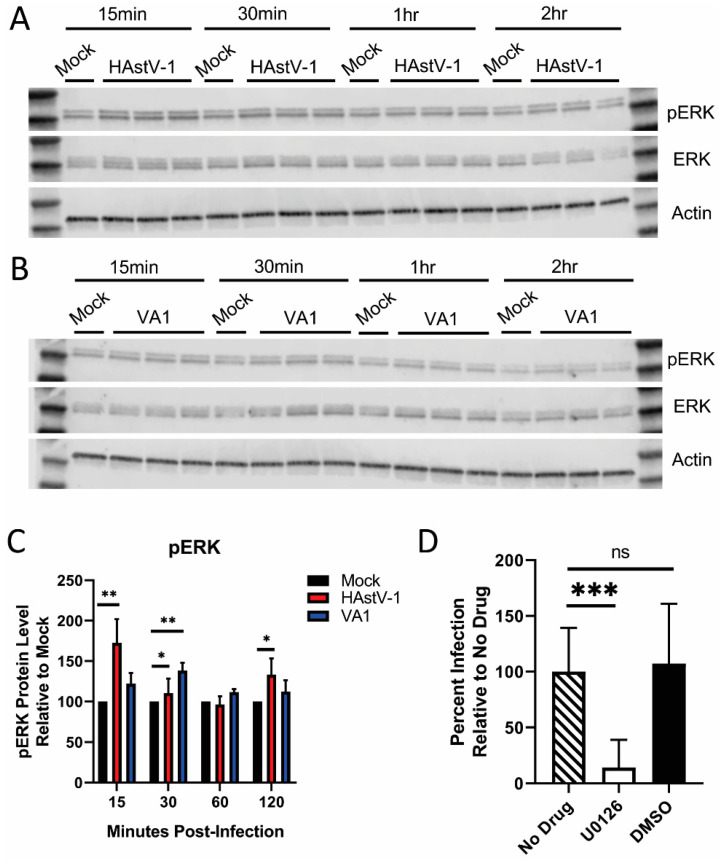
ERK1/2 is activated by VA1 and required for productive replication. Lysates from HAstV-1 (**A**) and VA1 (**B**) infected cells taken at the indicated times post-infection were blotted and probed for pERK, ERK, and β-actin. (**C**) Bands were then quantified by densitometry and normalized to β-actin then compared to mock-infection. (**D**) Caco-2 cells were pre-incubated 10 μM U0126 vehicle alone (DMSO) and then infected with VA1 (MOI of 2). Percent infection was measured by immunofluorescent staining for VA1 capsid at 24 hpi. Error bars indicate standard deviations from two independent experiments performed in triplicate, and asterisks show statistical significance as measured by ordinary one-way ANOVA as follows: *, *p* < 0.05; **, *p* < 0.01; ***, *p*  < 0.001.

To further examine how the prolonged replication kinetics would impact known cellular changes associated with astrovirus infection, we first examined ERK1/2 activation. We previously demonstrated ERK1/2 activation occurs within 15 min post-HAstV-1 infection and is required for productive replication [36] (Figure 3A). While the kinetics was similar (15 min compared to 30 min), the magnitude of ERK1/2 activation was decreased in VA1 infected cells (Figure 3B,C). Like HAstV-1, VA1 replication is dependent on ERK1/2 activation. Inhibition of ERK1/2 with U0126 reduced VA1 levels by 86% compared to no drug and vehicle control alone (Figure 3D). 

**Figure 4:**
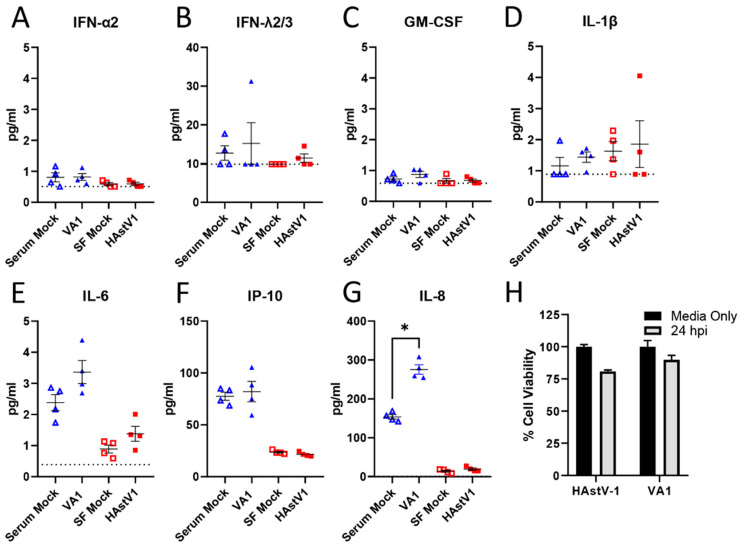
HAstV-1 and VA1 infection does not induce cytokines or cell death. A panel of 13 cytokines were analyzed from cell supernatants following 48 hpi for VA1 and serum-containing mock-infected wells and compared with supernatants from HAstV1 and serum free mock-infected wells 24 hpi. Levels of TNF-α, IL-12p70, IFN-β, IL-29, IL-10 and IFN-γ were all undetectable and are not shown. For the other 7 cytokines (**A**–**G**), the lower limit of detection is denoted by the dashed lines. Asterisks (*) denote statistical significance as measure by Mann Whitney U, with a cutoff value of *p* < 0.05. (**H**) Cell viability following VA1 and HAstV-1 infection at 24 hpi was measured using an ATP detection assay.

None of the astroviruses described to date induce inflammation or cell death *in vitro* or in vivo [35,41,42,43,44]. To determine if this was also true for VA1, we measured cytokine production in HAstV-1 and VA1 infected Caco-2 cells at the peak of infection (24 and 48 hpi respectively). From a panel of 13 cytokines, TNF-α, IL-12p70, IFN-β, IL-29, IL-10 and IFN- γ were undetectable in cell supernatants and minimal levels of IL-1β, IL-6, IFN-α2, GM-CSF, IFN-λ2/3 were detected (Figure 4A–E). IP-10 levels increased (Figure 4F); however, this was not significantly different from mock infection. In fact, only the pro-inflammatory cytokine IL-8 was significantly increased following VA1, but not HAstV-1 infection compared to mock infection (Figure 4G).

To assess cell death, Caco-2 cells were infected with HAstV-1 or VA-1 and cell death measured by ATP detection assay. Neither astrovirus strain induced significant cell death (Figure 4H). Combined, these studies highlight that VA1, similar to other astroviruses, fails to induce cell death or inflammation during infection [35,45]. Future studies are needed to define the role of IL-8 in VA1 pathogenesis. 

**Figure 5:**
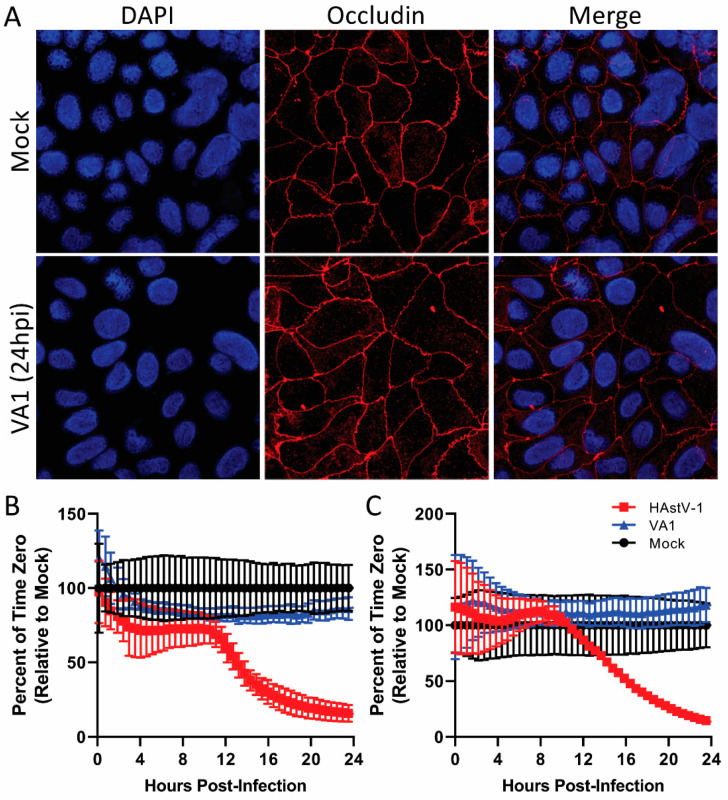
VA1 infection does not induce barrier permeability. (**A**) Representative images, taken at 60·magnification on Zeiss LSM 780 NLO, of Caco-2 monolayers infected with VA1 (MOI of 10) at 24 hpi show no disruption of occludin expression compared to mock-infected cells. Caco-2 monolayers grown on semi-permeable supports were infected with HAstV-1, VA1 at MOIs of 1 (**B**) and 10 (**C**), or mock-infected and transepithelial electrical resistance (TER) was measured from 0–24 hpi.

Despite the similarities, there are differences in the cellular responses to HAstV-1 and VA1 infection. We have previously shown that classical HAstVs increase epithelial barrier permeability [41,45]. In Caco-2 cells, this begins with the relocalization of the tight junction protein occludin from the cell periphery followed by a drop in transepithelial resistance (TER) resulting in increased barrier permeability [45]. VA1 infection had no impact on occludin relocalization even at an MOI of 10, nor did it impact TER (Figure 5). Extending the time of measurement up to 72 hpi also had no impact. In contrast, Caco-2 cells infected with HAstV-1 at an MOI of 1 or 10 showed a significant drop in TER beginning between 10–12 hpi (Figure 5B,C).

**Figure 6:**
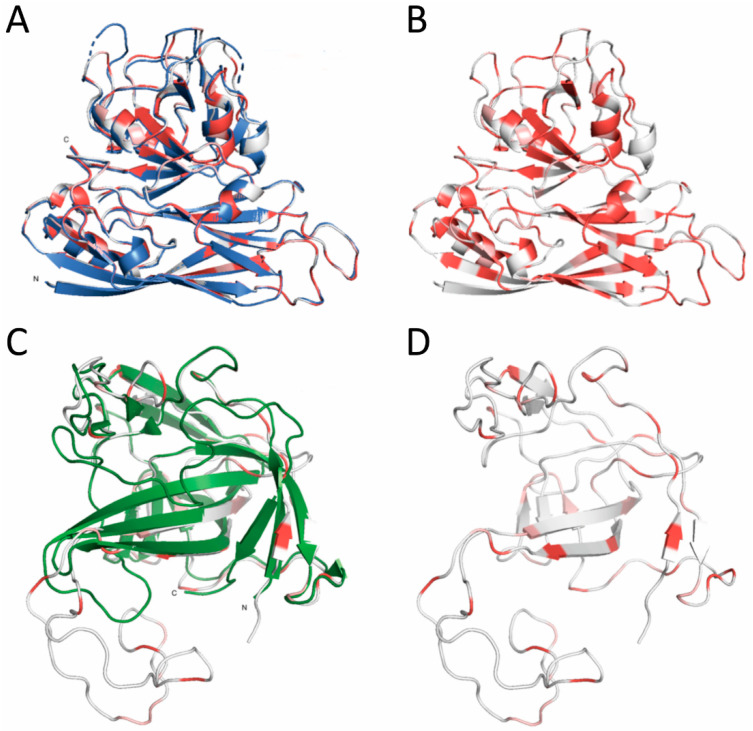
Alignment of classical HAstV-1 capsid structures with VA1 homology models. Crystal structure of the HAstV-1 (blue and green) aligned with homology models of VA1 capsid core (**A**) and spike (**C**). VA1 models (**B**,**D**) were marked with different colors based on its similarity with HAstV-1 reference: residues that are perfectly conserved (red), residues that are not perfectly conserved but that have a BLOSUM62 score higher than 0 (pink) and residues with a BLOSUM62 score lower than 0 or residues that are aligned to a gap in the reference (white).

Both HAstV and turkey astrovirus (TAstV) increased barrier permeability in vitro and in vivo [41,45] making VA1 the first identified astrovirus strain tested that does not impact permeability. We showed that the capsid protein alone is sufficient to increase permeability and relocalize occludin. Thus, we compared VA1 and HAstV-1 ORF2 genetic similarity at the nucleotide and amino acid levels. These viruses share 49.2% identity at the nucleotide level and 26.6% identity at the amino acid levels. Using publicly available protein sequences and published HAstV-1 structures, we overlaid the HAstV-1 capsid core and spike structures with that of the VA1. As predicted by the limited amino acid similarity, there were significant differences in the capsid tertiary structure (Figure 6A,C). While the capsid core for each virus shared 42.3% sequence identity, the spike domain only shared 11.9% identity (Figure 6B,D). Further studies are needed to understand how these differences impact receptor usage, viral replication kinetics and pathogenesis in vivo.

## 4. Discussion

VA1 was discovered during an outbreak of gastrointestinal illness in 2008. Although two of the six available samples from the outbreak were positive for VA1 infection, the simultaneous detection of HAstV-1 made it unclear which virus was associated with the outbreak [46]. Since its discovery, there has yet to be a study to identify a clear association with diarrheal illness. While previous studies have detected VA1 in cases of diarrhea, many studies do not screen for these novel genotypes making it difficult to definitively conclude whether VA1 is a major causative agent. Until recently, we were unable to even study VA1 in vitro.

In 2018, Janowski, et al. demonstrated that VA1 could be propagated in cell culture in numerous cell lines and more recently in enteroids [31,32,47]. This work was invaluable and allowed studies to compare VA1 to classical HAstV strains, which have been the primary focus of human astrovirus research. Taking advantage of this knowledge, we focused our studies on exploring VA1 and HAstV-1 similarities and differences in Caco-2 cells. Our studies highlight that VA1 and HAstV-1 share some features but do have distinct cellular impacts. Of interest is the apparent delay in replication kinetics. 

Here, we show clear differences in replication kinetics with VA1 having a delay of 6 h for dsRNA formation and capsid expression. This is not unique to Caco-2 cells. Previous studies have identified that optimal VA1 production does not occur until 96 hpi [34]. This is not due to cell death. We showed that VA1 does not induce significant cell death at any time point, which is consistent with published reports [31,47] and with other astrovirus genotypes [35,45]. While the mechanisms underlying the delay require further study, our data suggest that the delay may not be at the binding/entry step given the kinetics of ERK1/2 activation. Like HAstV-1, VA1 infected Caco-2 cells have an early (15–30 min) increase in ERK1/2, which is required for productive replication. Future studies should focus on generating the tools to investigate the complete VA1 and HAstV replication cycle. 

Unlike HAstV strains and the turkey astrovirus-2 (TAstV-2) [41,45], VA1 had no impact on barrier permeability or reorganization of cell junction proteins. This was true regardless of MOI (Figure 5) or time post-infection. Even at the peak of infection (48–96 hpi), VA1 failed to increase the epithelial barrier. This could be due to the differences in the capsid structure since HAstV-1 and TAstV-2 capsid proteins alone can induce these changes [41,45]. Alternatively, the VA1-induced increase in IL-8 (Figure 4) could be involved. 

Astrovirus infection is not associated with increased inflammation in vitro or in vivo [35,41,42,43,44]. Yet, in vitro, VA1 infection at a high MOI significantly increased the production of IP-10 and IL-6 in primary astrocytes and immortalized glial cells and IL-8 in primary astrocytes [47] and our studies show increased IL-8 expression. The contrast in the levels of IL-8 induction seen in our studies and in previous reports may be due to differences in MOI used or the dynamic range of detection between detection assays. These findings highlight that VA1 may drive a different cellular response depending on the cell tropism. IL-8 is an important inflammatory marker in the gut [43]. We know that inflammatory cytokines, particularly TNF-α, are associated with increased barrier permeability [48]. However, there are no reports on the role of IL-8 in intestinal barrier permeability. One study demonstrated that anti-IL-8 antibodies reduced acid-induced increase in alveolar epithelial permeability restoring alveolar fluid clearance to normal [49]. Future studies are needed to investigate the role of IL-8 in astrovirus pathogenesis. 

Human astroviruses, particularly VA1, can cause systemic infections, including encephalitis and viremia, highlighting the need for effective anti-viral therapies. Our studies demonstrate that NTZ inhibits VA1 replication even when administered up to 12 h post-infection highlighting the utility of NTZ as a broad-spectrum antiviral [50,51,52,53,54,55,56]. The work by Janowski et al. demonstrating that ribavirin and favipiravir also blocks infection [57] now gives clinicians an arsenal of options to treat astrovirus infections. 

In summary, our studies highlight the similarities and differences between classical HAstV-1 and non-classical VA1 infection in intestinal epithelial cells. These studies build upon the limited data of the cellular mechanisms of pathogenesis related to astrovirus infection. However, much more work is needed. We and others are creating the tools to eventually be able to explore the intricacies of astrovirus infection in vitro and in vivo.

## Data Availability

All relevant data are within the paper.
